# A Chloroform Fraction Derived from *Vitis vinifera* Root Ethanol Extract Attenuates Lipopolysaccharide-Induced Inflammatory Responses and Cognitive Dysfunction in BV-2 Microglia Cells and C57BL/6J Mouse Model

**DOI:** 10.3390/ijms26073126

**Published:** 2025-03-28

**Authors:** Yon-Suk Kim, Sang-Bong Lee, Shin-Il Kang, Woo-Jung Kim, Dong-Kug Choi

**Affiliations:** 1 Department of Biotechnology, Research Institute of Inflammatory Disease (RID), College of Biomedical and Health Science, Konkuk University, Chungju 27478, Republic of Korea; kimyonsuk@kku.ac.kr; 2 Department of Applied Life Sciences, Graduate School, BK21 Program, Konkuk University, Chungju 27478, Republic of Korea; 3 Biocenter, Gyeonggido Business and Science Accelerator, Gwanggyo-ro 147, Yeongtong-gu, Suwon 16229, Republic of Korea; wj0504@gbsa.or.kr

**Keywords:** neuroinflammation, microglia activation, *Vitis vinifera* root extract, Lipopolysaccharide (LPS), cognitive dysfunction

## Abstract

This study aimed to investigate the inhibitory effect of the chloroform fraction (CF) from *Vitis vinifera* root extract on LPS-induced neuroinflammation in BV-2 microglia cells and a C57/BL6J mouse model. CF significantly suppressed LPS-induced proinflammatory cytokines, including nitric oxide (NO), tumor necrosis factor-α (TNF-α), and interleukin-6 (IL-6) in BV-2 microglia cells. Mechanistically, CF inhibited LPS-induced activation of nuclear factor-κB (NF-κB) by blocking the p65 subunit and preventing the phosphorylation of NF-kappa-B inhibitor α (IκBα), while its effect was independent of the mitogen-activated protein kinase (MAPK) pathway. Furthermore, CF modulated the TRIF signaling pathway by regulating TANK-binding kinase 1 (TBK1) and interferon regulatory factor 3 (IRF3), which contributed to the suppression of inflammatory mediators in BV-2 microglia cells. In vivo, we evaluated the neuroprotective effects of CF against cognitive dysfunction and inflammatory responses in an LPS-induced mouse model. Our behavioral assessments, including the Morris water maze and Y-maze tests, demonstrated that CF alleviated LPS-induced spatial learning impairment and cognitive decline. Additionally, CF significantly reduced the levels of inflammatory cytokines in serum and inflammatory mediators proteins expression in whole brain in LPS-injected mice, suggesting a direct link between reduced inflammatory responses and improved cognitive function. These findings suggest that CF from *V. vinifera* root extract may serve as a potential therapeutic strategy for neurodegenerative diseases mediated by microglial activation, such as Alzheimer’s disease.

## 1. Introduction

Neuroinflammation is an immune response that occurs in the central nervous system (CNS) of the brain, and involves microglia, astrocytes, and neurons [1]. Additionally, neuroinflammation is known to be an important risk factor involved in the pathophysiological mechanisms of many neurological disorders, including trauma [2], brain infection [3], and age-related neurodegenerative diseases (NDDs) [4]. Neuroinflammation has been identified as a major cause of cognitive impairment and NDDs, such as Alzheimer’s disease (AD), Parkinson’s disease (PD), Huntington’s disease, multiple sclerosis (MS), and amyotrophic lateral sclerosis [5]. Lipopolysaccharide (LPS) is an endotoxin present in the outer membrane of Gram-negative bacteria that reaches the brain through nerve conduction and directly affects the CNS [6]. Systemic injection of bacterial LPS into mice induces disease by triggering an innate inflammatory response [7]. LPS-injected animal models are widely used to study mental and cognitive responses, systemic inflammation, and neuroinflammation [8]. This study used LPS to induce microglial activation-mediated neuroinflammatory reactions in vitro and cognitive impairment in vivo models.

Microglia, resident brain macrophages, have been shown to play a critical role in the initiation and development of neuroinflammation [9]. Microglia respond immediately to any changes to defense against pathogenic invasion and infection in the CNS [10]. Microglial cells play a central role in inducing neuroinflammation by activating toll-like receptor 4 (TLR4) in response to endotoxins [11]. BV-2 microglia cells are a mouse cell line used to study microglia function and neuroinflammation. This study used LPS-induced BV-2 microglia cells as a neuroinflammation model. Neuroinflammation is characterized by microglial activation and increased levels of inflammatory mediators such as cytokines, NO, reactive oxygen species (ROS), and other neurotoxic substances [12]. The mechanism of signal transduction via LPS-stimulated TLR4 is well characterized. There are two possible TLR4 signaling pathways activated by LPS: the myeloid differentiation primary response 88 (MyD88)-dependent and MyD88-independent (Toll/interleukin (IL)-1R domain-containing adapter-induced interferon (IFN)-β, TRIF-dependent), responsible for the expression of proinflammatory cytokines [13,14]. Furthermore, MAPKs are involved in LPS-induced expression of inflammatory mediators and NF-κB activation [14].

Throughout history, the use of phytotherapeutic agents for health-related purposes has been widespread, and they are regarded worldwide as an invaluable source of bioactive compounds that can help mitigate various diseases. The increased effectiveness of medicinal plants’ pharmacological properties has positioned traditional herbal medicine as a promising area for future medical practice in healthcare management. The scientific community’s interest in phytotherapeutic agents has grown, given their remarkable abundance of natural biomolecules demonstrating diverse biological activities. One of these medicinal plants is the grape. The grape (*Vitis vinifera*) is a perennial fruit and a well-known grape species from the *Vitaceae* family, specifically the *Vitis* genus [15]. Numerous studies indicate that the roots, stems, canes, leaves, seeds, fruits, pomace, and skin of *V. vinifera* contain various phytochemical compounds that enhance their antioxidant, antibacterial, anti-inflammatory, and anticancer characteristics [16,17,18,19,20,21,22,23,24,25]. However, according to previous comparative reports, the root of *V. vinifera* may be highly enriched with specific stilbenoids, which exhibit potential health benefits, including antioxidant and anti-inflammatory properties [26]. Despite these findings, most studies of the pharmacological effect of *V. vinifera* have been conducted using extracts from seeds, fruits, and stems, and studies using *V. vinifera* root extracts are very limited [27,28,29,30,31].

In particular, *V. vinifera* roots have long been used as a folk medicine in East Asia to treat pain, swelling, urinary problems, and fractures of muscles and bones [32]. However, despite its long history of use as a medicinal herb in oriental medicine, *V. vinifera* roots have not been studied sufficiently. Since these traditional medical uses have not been sufficiently scientifically verified, it is believed that experimental validation will provide novel insights and increase the validity of the study.

Thus, we primarily aim to investigate the anti-neuroinflammatory effect of *V. vinifera* root against LPS-induced BV-2 microglia cells and C57BL/6J amnesic mouse models. In this study, we fractionated *V. vinifera* root extract into five fractions using organic solvents and selected the fraction with the most potent inhibitory effect on NO production. Using the chloroform fraction (CF), we investigated its neuroprotective and anti-neuroinflammatory effect against LPS-induced BV-2 microglia cells. Afterward, we identified the active components and their contents in CF using liquid chromatography coupled with mass spectrometry (LC-MS). Further investigated was the protective role of CF against cognitive dysfunction induced by LPS in a C57BL/6J amnesic model. Our data suggests that the CF may suppress LPS-induced microglial activation and inflammatory responses and improve spatial learning and cognitive function.

## 2. Results

### 2.1. In Vitro Study

#### 2.1.1. Effects of *Vitis vinifera* Extracts and Fractions on NO Production and Cell Viability

**Figure 1:**
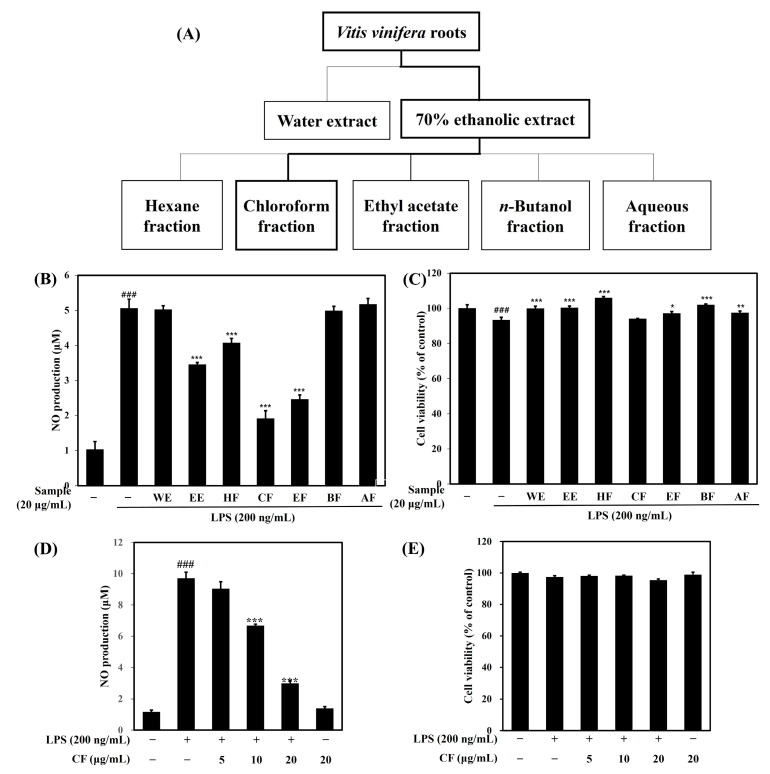
Scheme of the fractionation of the *V. vinifera* 70% ethanol extract using organic solvent partitioning (**A**). Effects of the crude *V. vinifera* extract and its fractions on NO production (**B**,**D**) and cell viability (**C**,**E**) in LPS-induced BV-2 microglia cells. BV-2 microglial cells were treated with crude ethanol of *V. vinifera* root extract and its fractions (20 μg/mL). Data are presented as mean ± SD from three independent experiments. ^###^ *p* < 0.001 versus control. * *p* < 0.05, ** *p* < 0.01, *** *p* < 0.001 versus LPS. WE; water extract, EE; 70% ethanol extract, HF; Hexane fraction, CF Chloroform fraction, EF; Ethyl acetate fraction, BF; *n*-Butanol fraction, AF; Aqueous fraction.

Preliminary results showed that the ethanol extract was more effective in inhibiting NO production than the aqueous extract, and then five fractions (HF; hexane fraction, CF; chloroform fraction, EF; ethyl acetate fraction, BF; *n*-butanol fraction, AF; aqueous fraction) were prepared using the ethanol extract (Figure 1A). The NO production inhibition effect was compared using seven samples at a concentration of 20 μg/mL. The NO inhibition effect was strong in the following order (CF (*p* < 0.001) > EF (*p* < 0.001) > EE (*p* < 0.001) > HF (*p* < 0.001) > BF > WE > AF) (Figure 1B). CF inhibited NO production by 77.5% compared to LPS, EF by 65%, EE by 42.3%, and HF by 25%. EF, EE, and HF strongly inhibited NO production, and CF showed the strongest NO production inhibition effect.

Cytotoxicity evaluation was performed at the same concentration, and compared to the control group, cell viability was more than 95% in all groups, and no cytotoxicity was observed (Figure 1C). Subsequent experiments were performed using only CF, which showed strong NO inhibition efficacy. We found that exposure of BV-2 microglia cells to CF (5–20 μg/mL) dose-dependently inhibited LPS-induced NO production (Figure 1D). In addition, no toxicity occurred when CF was treated alone without LPS, confirming that CF did not affect NO production under these conditions (Figure 1E).

#### 2.1.2. Effects of the CF on iNOS and COX-2 Protein Expression

**Figure 2:**
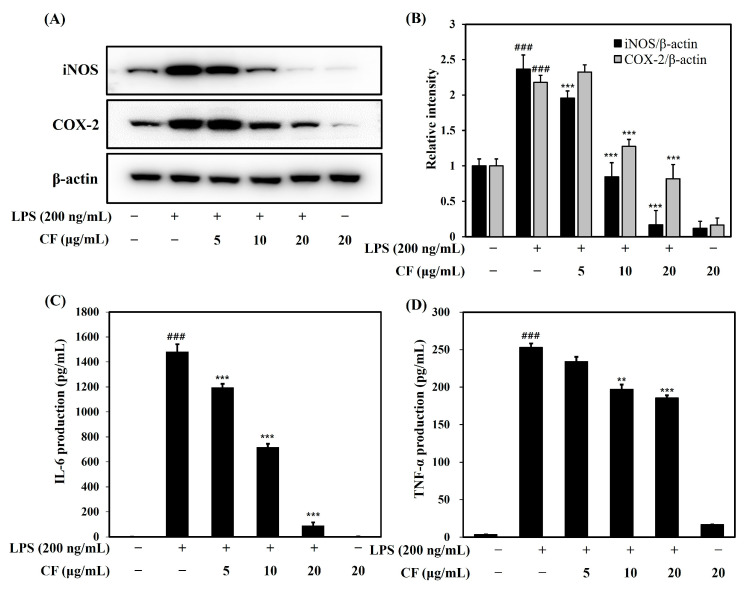
Effects of the CF on inflammatory-related proteins iNOS and COX-2 expression (**A**,**B**) and proinflammatory cytokines (IL-6 and TNF-α) production (**C**,**D**) in LPS-induced-BV-2 microglia cells. BV-2 microglia cells were pretreated for 1 h with the indicated concentrations of CF, followed by stimulation with LPS (200 ng/mL) for 18 h (**A**,**B**). Cell lysates were then harvested, and protein levels of iNOS and COX-2 were determined by Western blot. The production of IL-6 and TNF-α was measured using ELISA. The BV-2 microglia cells were pretreated for 1 h with the indicated concentrations of the CF before stimulation with LPS (200 ng/mL) for 18 h (IL-6) and 6 h (TNF- α). Data are presented as mean ± SD from three independent experiments. ^###^ *p* < 0.001 versus control and ** *p* < 0.01, *** *p* < 0.001 versus LPS.

The anti-neuroinflammatory effect of CF on the LPS-induced inflammation protein (iNOS and COX-2) was examined by a Western blot. Treatment with LPS significantly increased iNOS and COX-2 protein expression compared to the control group (Figure 2A,B, *p* < 0.001). However, pretreatment with the CF caused a dose-dependent decrease in the expression of these proteins. Therefore, the CF could regulate anti-neuroinflammatory effects by suppressing iNOS and COX-2 expressions.

#### 2.1.3. Effects of the CF on Inflammatory Cytokines

We examined whether CF affects inflammatory cytokines using ELISA. In response to LPS stimulation, macrophages release proinflammatory cytokines, such as TNF-α and IL-6. Our experiments showed that CF significantly inhibited LPS-induced IL-6 and TNF-α production in BV-2 microglia cells in a dose-dependent manner (Figure 2C,D, *p* < 0.01, *p* < 0.001). Therefore, it was confirmed that CF has strong neuroinflammation suppression efficacy by significantly suppressing NO and cytokines, which are inflammation-related mediators.

#### 2.1.4. Effects of the CF on MAPK Protein Expression

**Figure 3:**
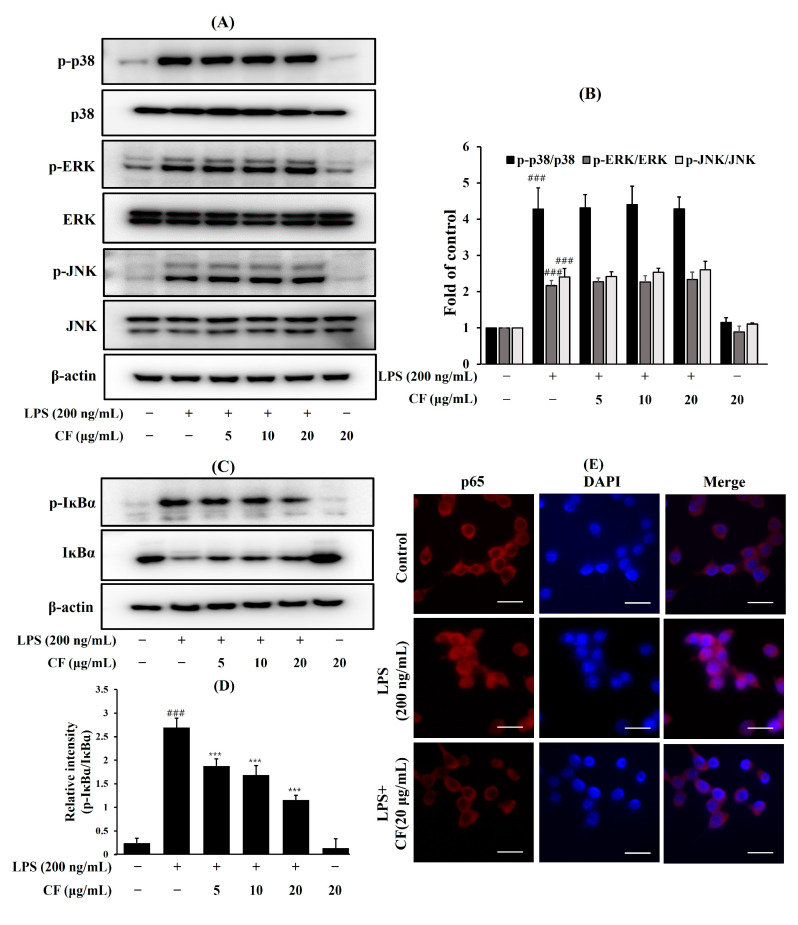
Effect of CF on MAPKs protein expression (**A**,**B**), phosphorylation of IκB (**C**,**D**), and nuclear translocation of the NF-ĸB (**E**) in the LPS-induced BV-2 microglia cells. The BV-2 microglia cells were pretreated for 1 h with the indicated concentrations of the CF before stimulation with LPS for 30 min (**A**,**B**) and 15 min (**C**,**D**). Localization of NF-κB p65 was visualized using a fluorescence microscope after immunofluorescence staining with anti-NF-κB p65 antibodies (red) (**E**). The cells were stained with DAPI to visualize the nuclei (blue)—scale bar: 50 μm. Data are presented as mean ± SD from three independent experiments. ^###^ *p* < 0.001 versus control, *** *p* < 0.001 versus LPS.

To further investigate the mechanisms underlying the anti-inflammatory effects of the CF, we examined its effects on the phosphorylation of MAPKs (ERK1/2, JNK, and p38). Interestingly, our results showed that pretreatment with the CF did not suppress the phosphorylation of ERK, p38, and JNK (Figure 3A,B). Meanwhile, as shown in Figure 3C,D, the CF decreased the phosphorylation levels of IκBα. In addition, LPS induced translocation of p65 into the nucleus in BV-2 microglia cells, but pretreatment with the CF decreased this translocation, as shown by immunocytochemistry staining (Figure 3E). Given that the CF inhibited the LPS-induced degradation of IκBα, we concluded that NF-κB p65 translocation from the cytosol to the nucleus was possible.

#### 2.1.5. Effects of the CF on TBK1 and IRF3 Protein Expression

**Figure 4:**
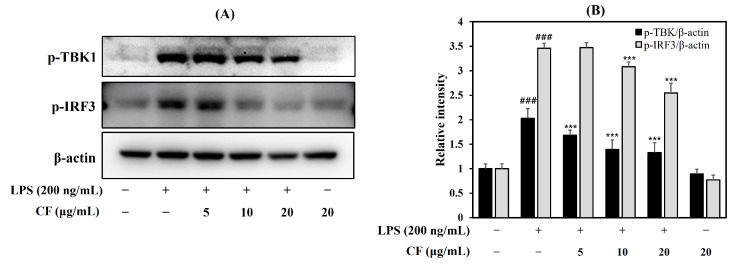
Effect of CF on phosphorylation of TBK1 and IRF3 (**A**,**B**) in the LPS-induced BV-2 microglia cells. The BV-2 microglia cells were pretreated for 1 h with the indicated concentrations of the CF before stimulation with LPS (200 ng/mL) for 1.5 h. Cell lysates were then harvested, and protein levels were determined by Western blot. Data are presented as mean ± SD from three independent experiments. ^###^ *p* < 0.001 versus control, *** *p* < 0.001 versus LPS.

To understand the mechanisms by which the CF regulates the TRIF signaling pathway, we investigated the phosphorylation of TBK1 and IRF3 using Western blotting. The results showed that LPS stimulated the phosphorylation of TBK1 and IRF3 (Figure 4A,B, *p* < 0.001). In contrast, the CF decreased TBK1 and IRF3 expression dose-dependently (*p* < 0.001). This suggests that the anti-neuroinflammatory activity of CF occurs through suppression of the TRIF signaling pathway.

#### 2.1.6. LC-MS and HPLC Analysis of CF

Compounds were identified from CF using LC-MS. Five compounds were identified from CF by searching available natural product databases online and in-house MS/MS spectral libraries using high-resolution mass and MS/MS spectral data. Data concerning the compound identification is shown in Table 1. We were unable to quantify all five compounds. However, among the five components, ethyl caffeate focuses on one of the main components of CF, and its content was subsequently measured using HPLC. The ethyl caffeate content in CF was analyzed using HPLC, which was 5.95 ± 0.33 μg/mg.

#### 2.1.7. Anti-Neuro Inflammatory Effects of Ethyl Caffeate

**Figure 5:**
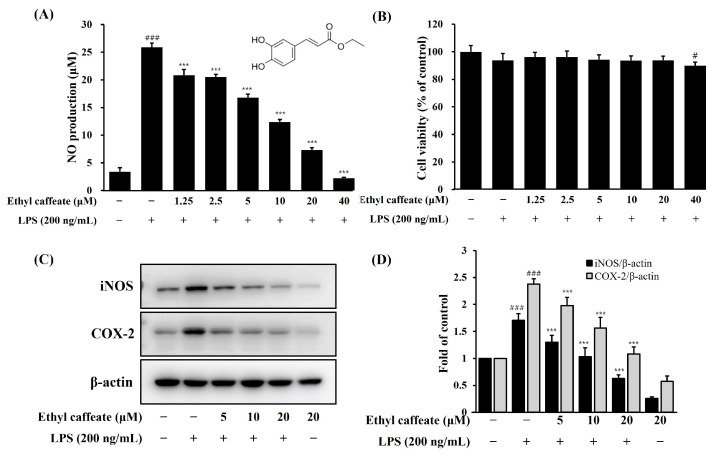
Effects of the ethyl caffeate on NO production (**A**), cell viability (**B**), and inflammatory protein iNOS and COX-2 expression (**C**,**D**) in LPS-induced BV-2 microglia cells. BV-2 microglia cells were pretreated for 1 h with the indicated concentrations of ethyl caffeate, followed by stimulation with LPS (200 ng/mL) for 18 h (NO assay (**A**)) and 24 h (MTT assay (**B**)). BV-2 microglia cells were pretreated with ethyl caffeate for 1 h and then stimulated with LPS (200 ng/mL) for 18 h (**C**,**D**). Cell lysates were then harvested, and protein levels of iNOS and COX-2 were determined by Western blot. Data are presented as mean ± SD from three independent experiments. ^#^ *p* < 0.05, ^###^ *p* < 0.001 versus control, *** *p* < 0.001 versus LPS.

The anti-neuroinflammatory effect of ethyl caffeate, one of the main components of CF, was analyzed. At concentrations ranging from 1.25 to 40 μM, NO production decreased in a dose-dependent manner (Figure 5A, *p* < 0.001). Since cell viability decreased at 40 μM ethyl caffeate (Figure 5B), subsequent experiments were performed at a nontoxic concentration of less than 20 μM. iNOS and COX-2 protein levels were confirmed using Western blot. Our results show that ethyl caffeate reduces LPS-induced iNOS and COX-2 protein expression dose-dependently in BV-2 microglia cells (Figure 5C,D, *p* < 0.001). Among the various components of CF, we focused on ethyl caffeate and determined that ethyl caffeate is one of the main components of CF with strong anti-neuroinflammatory effects.

### 2.2. In Vivo Study

#### 2.2.1. The Effects of CF and LPS on MWM Test and Y-Maze Test

**Figure 6:**
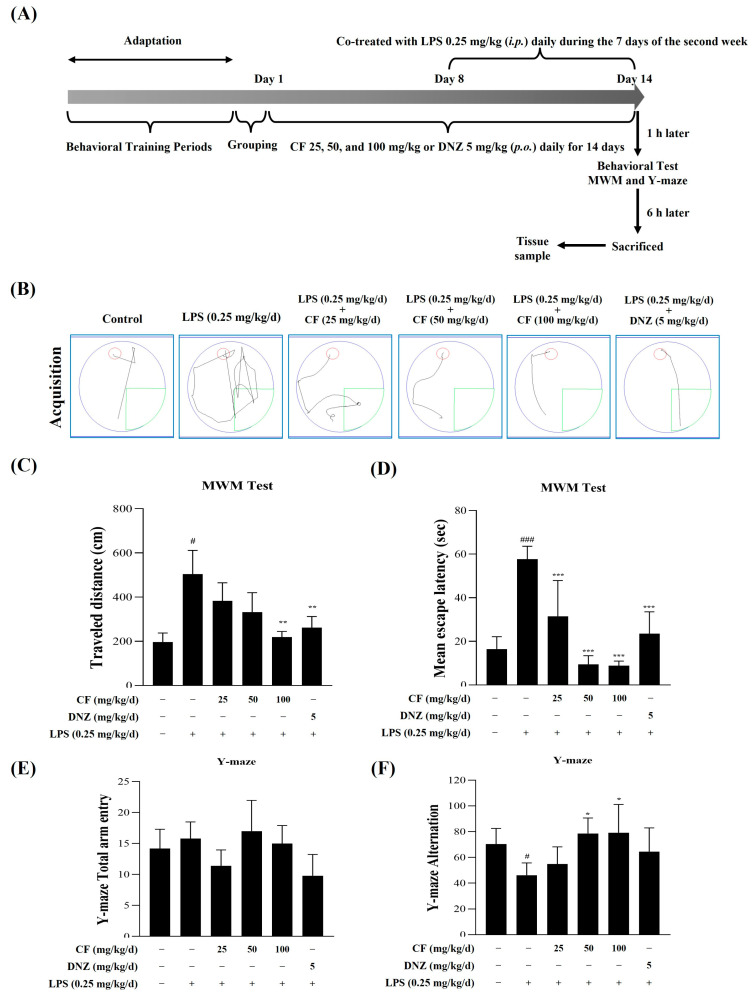
The neuroprotective effects of CF on LPS-induced cognitive dysfunction were evaluated using the MWM and Y-maze tests. We administered CF (25, 50, and 100 mg/kg/d; p.o.) or donepezil (DNZ; 5 mg/kg/d; p.o.) for 14 days and LPS (0.25 mg/kg/d; i.p.) for 7 days starting from the eighth day, as shown in the overall experimental schedule (**A**). The trajectories depict the path length covered by the mice (**B**). The total distance traveled (**C**) and the escape latency to locate the hidden goal (**D**) were recorded during the MWM test. The total number of arm entries (**E**) and the percentage of spontaneous alternations (**F**) were measured for the Y-maze. Data are expressed as mean ± SD (n = 6 per group). ^#^ *p* < 0.05 and ^###^ *p* < 0.001 indicate comparisons between the LPS-treated and control groups; * *p* < 0.05, ** *p* < 0.01 and *** *p* < 0.001 indicate comparisons between groups treated with different concentrations of CF and DNZ versus those treated with LPS alone.

To investigate the effects of CF on spatial learning and memory, we analyzed behavioral tests, including the MWM and Y-maze tests. For 14 days, we administered CF (25, 50, and 100 mg/kg/d; p.o.) or donepezil (DNZ, 5 mg/kg/d; p.o.). During the 7 days of the second week, we administered LPS (0.25 mg/kg/d; i.p.). Behavioral tests were conducted 1 h after the administration of CF (25, 50, and 100 mg/kg/d; p.o.) or DNZ (5 mg/kg/d; p.o.), as well as after the administration of LPS (0.25 mg/kg/d; i.p.) on the last day, Day 14 (Figure 6A). Behavioral tests were conducted in sequence, starting with the MWM test, followed by a rest period for the mice, and then the Y-maze test.

We observed no differences in escape latency and distance traveled to reach the platform across all groups during the training period. Our result showed that the administration of LPS (0.25 mg/kg/day; i.p.) alone provides higher escape latency time and traveled distance to reach the platform in comparison to the control group (Figure 6B–D, ** *p* < 0.01 and *** *p* < 0.001). However, the levels of escape latency time and traveled distance to reach the platform were significantly lower in the CF (25, 50, and 100 mg/kg) treatment groups compared to the LPS group. The LPS-treated group did not remember the platform location compared to the control group, but the DNZ-treated group, which was used as a positive control, took less time to find the platform than the LPS-treated group.

Regarding the behavioral performance of mice in the Y-maze task, we observed a significant group difference in spontaneous alternation. In the LPS-treated group, spontaneous alternation in the Y-maze was reduced, indicating working memory impairment. CF (25, 50, 100 mg/kg/d; p.o.) and DNZ (5 mg/kg/d; p.o.) treatment groups significantly enhanced the LPS (0.25 mg/kg/d; i.p.)-induced decrease spontaneous alternation (Figure 6E,F, *p* < 0.05). However, it was confirmed that CF (25 mg/kg/d; p.o.) did not show statistical significance. Therefore, the results of the MWM and Y-maze tests confirmed a significant memory improvement effect in the CF treatment and donepezil (DNZ, 5 mg/kg/day; p.o.) treatment groups.

#### 2.2.2. The Effects of CF Against LPS on Serum IL-6 and TNF-α Levels

**Figure 7:**
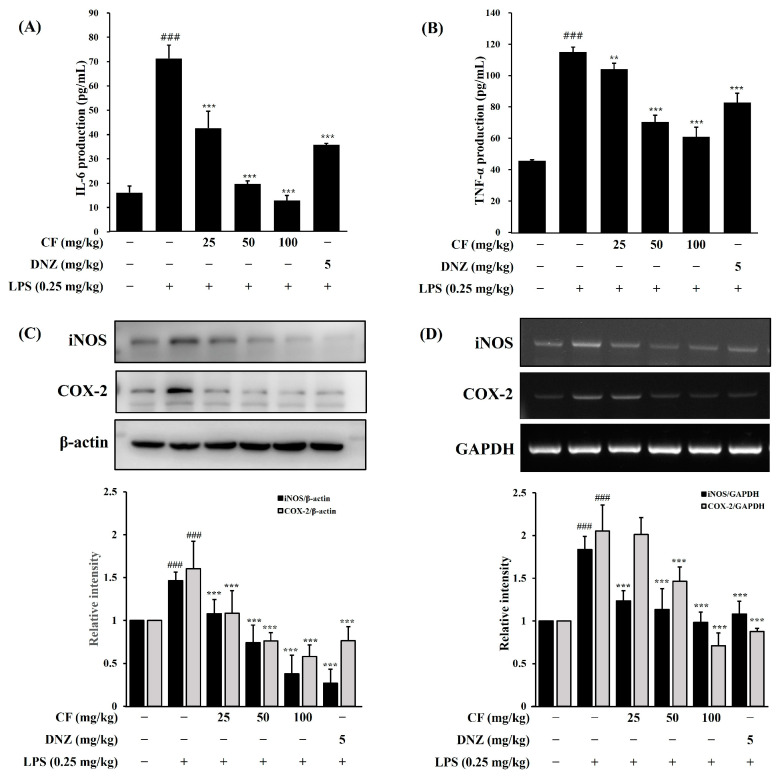
The effects of CF on serum proinflammatory cytokines IL-6 and TNF-α levels in LPS-induced cognitive dysfunction. Detection of IL-6 (**A**) and TNF-α (**B**) in serum by ELISA. Effects of CF on whole brain iNOS and COX-2 protein and mRNA expression levels in LPS-induced cognitive dysfunction (**C**,**D**). Data are expressed as SD (*n* = 3 per group). ^###^ *p* < 0.001, LPS-treated group vs. control group; ** *p* < 0.01 and *** *p* < 0.001: groups treated with different concentrations of the CF and DNZ/LPS vs. group treated with LPS alone. LPS (0.25 mg/kg/d; i.p), CF (25, 50, and 100 mg/kg/d; p.o.) and donepezil (DNZ, 5 mg/kg/d; p.o.).

In the group that received LPS (0.25 mg/kg/d; i.p), the levels of inflammatory cytokines (IL-6 and TNF-α, *p* < 0.001) were dramatically increased in the serum, in comparison to the control group (Figure 7A,B). Interestingly, our findings showed that the groups receiving the dose of 25, 50, and 100 mg/kg/d of CF have lower levels of inflammatory cytokines (IL-6 and TNF-α, *p* < 0.01, *p* < 0.001) in the serum compared to the LPS-treated group. The positive control group, DNZ, also had lower inflammatory cytokine levels than the LPS treatment group. The CF groups showed a strong inhibitory effect on serum inflammatory cytokines (IL-6 and TNF-α) dose-dependently, and these results suggest that CF has an excellent inhibitory effect on inflammatory cytokines increased by LPS.

#### 2.2.3. The Effects of CF Against LPS on Whole Brain iNOS and COX-2 Levels

Compared to the control group, the LPS-treated group notably increased the iNOS and COX-2 protein expression (Figure 7C, *p* < 0.001) in whole brain homogenates. On the other hand, the CF (25, 50, and 100 mg/kg/d, *p* < 0.001) and donepezil (*p* < 0.001) significantly suppressed the iNOS and COX-2 protein expression compared to the LPS-treated group.

Moreover, RT-PCR detected the iNOS and COX-2 mRNA expression levels in the whole brain homogenates. The LPS treatment group significantly increased iNOS and COX-2 gene expression levels compared to the control group (Figure 7D, *p* < 0.001). In contrast, CF treatment groups (25, 50, and 100 mg/kg/d, *p* < 0.001) and donepezil group (*p* < 0.001) show that the gene expression level of iNOS and COX-2 were markedly down-regulated in comparison to the LPS-treated group. We confirmed that CF inhibited iNOS and COX-2 expressions compared to the LPS-treated group at both protein and RNA levels, confirming that CF has anti-neuroinflammatory efficacy.

## 3. Discussion

The activation of microglial cells plays an important role in initiating, maintaining, and resolving inflammatory processes and is involved in developing neurodegenerative diseases [33,34]. Microglia are the primary immune cells of the central nervous system, and their primary function is to defend the host by destroying invasive pathogens, promoting tissue repair, and maintaining tissue homeostasis through their influence on surrounding astrocytes and neurons [35]. From this perspective, neuroinflammation is intended to protect the central nervous system. However, prolonged and unchecked activation of microglia can lead to the overproduction of various neuroinflammatory factors, including proinflammatory cytokines such as TNF-α, IL-6, and IL-1β, as well as free radicals, ultimately exacerbating neuronal damage [36]. 

Roots are known to be important sites for producing secondary metabolites in response to environmental stress (e.g., microbial infection, physical damage), and thus are likely to contain unique bioactive compounds. In particular, roots have been reported to produce specific stilbenoids and other bioactive molecules in response to environmental stimuli, which are not commonly found in other parts of the plant. Therefore, by studying the roots, we have the potential to discover previously unreported or relatively less studied bioactive substances in *Vitis vinifera*. This can serve as fundamental data for developing novel functional materials. Furthermore, to the best of our knowledge, there have been no previous reports on the anti-neuroinflammatory effects and cognitive function improvement associated with *Vitis vinifera* root extract. This study is the first to explore these effects, highlighting its novelty and potential contribution to the field. This study aimed to demonstrate the efficacy of *V. vinifera* root extract and its fractions in regulating neuroinflammation.

NO is produced by iNOS and is involved in promoting inflammatory responses. Excessive production of this mediator by activating microglia further damages neurons, contributing to the progression of neurodegenerative diseases [37,38]. Thus, inhibiting these inflammatory mediators is a promising therapeutic strategy against microglia activation-mediated neuroinflammation. In this study, treatment with *V. vinifera* root extract and its fractions inhibited LPS-induced NO production in BV-2 microglia cells, with the chloroform fraction (CF) showing the most potent inhibitory effect. Moreover, CF treatment significantly reduced LPS-triggered iNOS and COX-2 expression and dose-dependently suppressed the secretion of inflammatory cytokines (TNF-α and IL-6).

Furthermore, LPS stimulates TLR4 to activate downstream MAPK signaling pathways, triggering microglial activation and the production of inflammatory mediators [39]. To clarify how the CF downregulated inflammatory mediator and cytokine production, we examined its effects on LPS-induced activation of the MAPK (JNK, p38, and ERK) pathways. Interestingly, the CF did not inhibit LPS-induced phosphorylation of JNK, ERK, or p38. Meanwhile, NF-κB is the primary transcription factor regulating most proinflammatory cytokines, including IL-6 and TNF-α, making the NF-κB signaling pathway a key target for anti-inflammatory interventions. Accordingly, we further examined whether the CF inhibited LPS-mediated NF-κB activation. A previous study reported that NF-κB is activated by phosphorylation of IκBα via MAPK activation, which plays an essential role in apoptosis, differentiation, and cell proliferation by transmitting various extracellular signals to the nucleus [40]. However, in the present study, the CF did not affect MAPK signaling but suppressed inflammation by inhibiting NF-κB activity by suppressing IκBα phosphorylation. Xie et al. reported similar findings with delavatine A, which reduced NO and proinflammatory cytokine production via the NF-κB pathway without interfering in MAPK pathways [41]. In our study, CF showed anti-inflammatory activity without affecting the MAPK pathway.

The binding of ligands to TLRs induces downstream signaling via two distinct pathways, the MyD88-dependent and TRIF-dependent, for the induction of proinflammatory cytokines and IFN-stimulated genes [42]. These two pathways collectively activate transcription factors such as NF-κB and members of the IRF family. TRIF mediates TLR4-dependent NF-κB and IRF3 activation, leading to cytokine production and type-I IFN [43]. IRF3 activation is driven by C-terminal phosphorylation of TANK-binding kinase 1 (TBK1) and inhibitor-κB kinase ε (IKKε), which interact with TRIF via a signalosome complex containing NAK-associated protein (NAP1) and TNF receptor-associated factor (TRAF3) [44]. Since kinases are often pivotal therapeutic targets, TBK1 is the most influential kinase in TRIF-dependent TLR signaling. Phosphorylated IRF3 forms dimers and translocates to the nucleus, where it binds to IFN-stimulated response elements in the promoter regions of genes such as IFNβ and IP-10 [45]. These IRF3-regulated genes are crucial for antiviral and antibacterial immune responses [46]. Our data suggests that the CF suppresses LPS-induced inflammatory mediators by inhibiting the NF-κB and TRIF-dependent signaling pathways without MAPK signaling.

Additionally, we analyzed the components of CF using LC-MS and quantified ethyl caffeate by HPLC. Previous studies have reported on the anti-inflammatory properties of ethyl caffeate [47]. The concentration of pure ethyl caffeate showing activity (1.25 to 20 µM) was higher than the concentration of ethyl caffeate (0.45 µM) delivered by CF (20 µg/mL), therefore suggesting that while ethyl caffeate may contribute to the activity of CF, other active compounds are also most likely present.

Although further mechanistic studies of ethyl caffeate are needed, our findings indicate that ethyl caffeate significantly suppressed inflammatory markers, including NO, iNOS, and COX-2, in LPS-induced BV-2 microglia cells. It was reported that vitisin A, one of the main components of *V. vinifera* root extract, showed excellent anti-inflammatory activity in LPS-induced RAW264.7 cells [48]. Therefore, it is thought that the anti-neuroinflammatory effect of *V. vinifera* root is due to complex components rather than the effect of ethyl caffeate alone. Further study requires quantifying each major component of *V. vinifera* roots and analyzing their individual compound and combined compound effects.

LPS injections in mice induce cognitive, learning, and memory impairments [49,50]. Intraperitoneal LPS injections are widely employed as a neuroinflammation model, as they elicit a systemic inflammatory response that can reach the central nervous system via the bloodstream. Accordingly, the Morris water maze (MWM) and Y-maze tests are commonly used to assess spatial working memory in such models. In this study, oral administration of CF (25, 50, and 100 mg/kg/day) significantly alleviated LPS-induced cognitive deficits in C57BL/6J mice, as evidenced by the MWM and Y-maze tests. Additionally, CF markedly decreased proinflammatory cytokine levels (IL-6 and TNF-α) in serum and inflammatory protein expression (iNOS and COX-2) in whole brain tissue in the LPS-induced neuroinflammation model.

There are various methods for analyzing inflammatory cytokines in animal models treated with LPS, such as measuring cytokines in serum, ELISA, RT-PCR, Western blotting, and immunofluorescence staining. Each of these methods has its own advantages, and an appropriate method can be selected depending on the specific research objective. Among these, ELISA is the most widely used method for measuring inflammatory cytokines in animal models due to its high accuracy and quantification capability, making it a reliable tool for assessing systemic inflammatory responses. In our lab, we utilized ELISA to analyze cytokines in serum, as serum cytokine levels serve as systemic inflammatory indicators that reflect the overall immune response induced by LPS administration. On the other hand, iNOS and COX-2 were measured in brain tissue to evaluate neuroinflammation specifically. Since iNOS and COX-2 are key markers of neuroinflammation, their expression levels provide direct insights into the inflammatory response occurring within the brain. Unlike cytokines, which can circulate systemically, iNOS and COX-2 are intracellular enzymes whose upregulation is closely associated with localized inflammation within specific tissues, making brain tissue the most appropriate sample for their measurement. Thus, our approach allowed us to distinguish between systemic inflammation (measured through cytokines in serum) and localized neuroinflammation (assessed through iNOS and COX-2 expression in brain tissue), providing a more comprehensive understanding of the inflammatory response induced by LPS.

Although further research is needed to elucidate the protective mechanisms of CF in the cortex and hippocampus of LPS-stimulated mice, our findings indicate that CF may improve cognitive impairment by exerting an anti-neuroinflammatory effect. In a previous study, extracts from *V. vinifera* fruit were reported to enhance cognition and memory in an aluminum-induced Alzheimer’s disease animal model [51]. In future studies, it would be interesting to investigate the memory-enhancing efficacy of CF using other cognitive impairment animal models.

## 4. Materials and Methods

### 4.1. Materials

Fetal bovine serum (FBS) and penicillin–streptomycin were purchased from Hyclone (Thermo Scientific, Waltham, MA, USA). LPS (*Escherichia coli* 055:B5) was obtained from Sigma Chemical Co. (St. Louis, MO, USA). Enzyme-linked immunosorbent assay (ELISA) kits for IL-6 and TNF-α were purchased from (R&D System, Minneapolis, MN, USA). Antibodies against iNOS, COX-2, p38, phosphor (p)-p38, ERK1/2, p-ERK1/2, JNK, and p-JNK were supplied by Santa Cruz Biotechnology Inc. (Dallas, TX, USA). Antibodies for beta-actin, IκB-α, p-IκB-α, and NF-κB p65 were provided by Cell Signaling Technology (Danvers, MA, USA). Polyvinylidene fluoride (PVDF) membranes were purchased from GE Healthcare Life Sciences (Amersham Hybond-P, Buckinghamshire, UK). Ethyl caffeate (CFN97136) was purchased from Chemfaces (Wuhan, Hubei, China).

#### 4.1.1. Plant Materials

Roots of *V. vinifera* were purchased from the Herbal Medicine Market (Jecheon, Korea). Prof. K. H. Leem (Department of Herbology, Semyung University) authenticated the plant sample, and voucher specimens (KUB20-02) were deposited in the Konkuk University Herbarium.

#### 4.1.2. Preparation of the Extract and Its Fractions

The roots of *V. vinifera* were extracted with water and 70% ethanol. The water extracts (dry weight, 100 g/1.0 L) were decocted for approximately 2 h one time, filtered, and then lyophilized. The roots of *V. vinifera* (dry weight, 5.3 kg) were soaked in 53 L of 70% aqueous ethanol for 72 h at room temperature (RT) and then filtered with Whatman No. 41. The mixture was filtered and concentrated using a rotary evaporator (EYELA, Tokyo, Japan). After evaporation of ethanol, the extracts were lyophilized using a freeze dryer (IlshinBioBase, Dongducheon, Republic of Korea). The aqueous concentrate (100 g/1.0 L in water) was placed in a separating funnel, and hexane was added to remove low-polar substances three times (1.0 L each time). The residual aqueous fraction was fractionated three times with chloroform (1.0 L each time), three times with ethyl acetate (1.0 L), and then three times with *n*-butanol (1.0 L) using a separating funnel. All solvent fractions were concentrated to remove the organic solvent, then water was added, frozen, and lyophilized. Extraction yields of water extract and 70% ethanol extract were 4.7 and 7.18% *w*/*w* of the original plant material, respectively. Yields of hexane, chloroform, ethyl acetate, *n*-butanol, and aqueous fractions were 2.48, 1.81, 9.18, 10.10, and 47.96% *w*/*w* of the 70% ethanol extract, respectively.

#### 4.1.3. Liquid Chromatography–Mass Spectrometry (LC-MS) Analysis

All LC/MS analyses were carried out using an Orbitrap exploris240 (Thermo Fisher Scientific, Waltham, MA, USA) coupled to a vanquish horizon UHPLC system (Thermo Fisher Scientific, Waltham, MA, USA). Chromatographic separation of metabolite was conducted using an ACQUITY UPLC^®^ BEH C18 column (2.1 × 150 mm, 1.7 μm, (Thermo Fisher Scientific, Waltham, MA, USA) and operated at 50 °C. The LC-MS system consisted of a heated electrospray ionization probe (HESI-II) as an ionization source. The HESI was operated at 350 °C with a spray voltage of 5.0 kV. The nebulizer sheath gas flow rate and auxiliary gas flow rate were set at 50 arb and 5 arb, respectively. Mass spectrometric analysis was performed in polarity switching ability, performing the MS/MS scan with the following parameters: *m*/*z* range 100–1000; collision-induced dissociation energy 35%; data-dependent scan mode. The Orbtrap analyzer was used to acquire high-resolution mass spectra data with a mass resolving power of 90,000 FWHM at *m*/*z* 400. The data-dependent tandem mass spectrometry (MS/MS) experiments were controlled using the menu-driven software (version, 4.1.50) provided by the Xcalibur system (Thermo Fisher Scientific, Waltham, MA, USA). The high-resolution mass and MS/MS spectral data were used to search for natural-product database available online using compound discoverer (Thermo Fisher Scientific, Waltham, MA, USA) and using NIST library (NIST/EPA/NIH Mass Spectral Library, Gaithersburg, MD, USA) and program (NIST Mass Spectral Search Program, Gaithersburg, MD, USA).

#### 4.1.4. High-Performance Liquid Chromatography (HPLC) Analysis

The comparative HPLC analysis of the CF and authentic ethyl caffeate confirmed that the chromatogram of the active peak matches that of ethyl caffeate. HPLC analysis was carried out to determine their contents according to the following conditions. HPLC (Ultimate3000, Thermo Scientific, Germering, Germany), Shimadzu Shim-pack C18 column (250 × 4.6 mm, 5 μm), and UV detector were used. Mobile phases were formed using 0.5% phosphoric acid in DW as eluent A and acetonitrile (ACN) as eluent B. The gradient programming used was as follows: 0–3 min, linear gradient from 0% to 40% B; 3–28 min, linear gradient from 40% to 45% B; 28–30 min, linear gradient from 45% to 100% B. The flow rate was 1.0 mL/min, and compounds were detected at 324 nm. The solvent was filtered through a 0.22 µm filter and degassed. The sample injection volume was 20 µL. The retention time for ethyl caffeate was 12.72 min. Qualification was performed from the peak area of the sample using the standard graph.

### 4.2. In Vitro Study

#### 4.2.1. Cell Culture and Cell Viability Assays

BV-2 microglia cells were cultured in DMEM medium containing heat-inactivated 5% fetal bovine serum, streptomycin (100 μg/mL), and penicillin (100 unit/mL) at 37 °C in a humidified incubator in a 5% CO_2_ atmosphere. Effects of crude ethanol extract of *V. vinifera* root and its fractions on BV-2 microglia cell viability were determined by MTT assay [52], which tests the normal metabolic status of cells based on the assessment of mitochondrial activity. BV-2 microglia cells were seeded in a 24-well plate at 5.0 × 10^4^ cells/well. After 24 h, the cells were treated with different concentrations of the samples (5–20 μg/mL of CF, 1.25–40 μM of ethyl caffeate) and incubated for 1 h and later incubated with or without LPS (200 ng/mL) for 24 h. MTT stock solution (0.5 mg/mL) was added and incubated for 2 h. The supernatants were aspirated, and the formazan crystals in each well were dissolved in 400 μL of DMSO. Absorbance was measured using a spectrometer at a wavelength of 540 nm. Cell viability was expressed as a percentage of the control and mean ± standard deviation.

#### 4.2.2. NO Assay

Accumulated nitrite released into the culture medium was measured to assess NO production. BV-2 microglia cells were seeded into a 6-well culture plate at 5 × 10^4^ cells/well density. After 24 h, cells were pretreated with different concentrations of the samples (5–20 μg/mL of CF, 1.25–40 μM of ethyl caffeate) for 1 h and incubated with LPS (200 ng/mL) for 18 h. The supernatant was collected, and NO production was determined using Griess reagent. The absorbance of the reaction mixtures was measured at 550 nm using a microplate reader (TECAN, Männedorf, Switzerland). Data are presented as mean ± SD.

#### 4.2.3. Measurement of the Levels of TNF-α and IL-6 by ELISA

The cell suspension was prepared by inoculating BV-2 microglia cells into a 6-well culture plate (5 × 10^5^ cells/well). After 24 h, cells were pretreated with different concentrations of the CF (5, 10, and 20 μg/mL) for 1 h and incubated with or without LPS (200 ng/mL) for 18 h (IL-6) or 6 h (TNF-α). The levels of TNF-α and IL-6 were measured in the collected supernatant of BV-2 microglia cells and mice serum using ELISA kits (Mouse IL-6 (DY406-05), Mouse TNF-α (DY410-05)) according to the manufacturer’s protocol (R&D System, Minneapolis, MN, USA). The absorbance was measured at 450 nm using a microplate reader. Data are presented as mean ± SD.

#### 4.2.4. Western Blotting

BV-2 microglia cells were seeded in 6-well plates and incubated for 24 h. The samples were treated differently depending on the proteins to be analyzed (iNOS and COX-2 for 18 h, MAPKs for 30 min, and p-TBK1 and p-IRF3 for 1 h 30 min). Cultured cells were lysed with a lysis buffer and collected for protein extraction.

Mouse whole brain tissue was homogenized using a bead mill homogenizer (Bead Ruptor 24^TM^, OMNI International, Kennesaw, GA, USA) with lysis buffer. The lysates were incubated on ice for 15 min and centrifuged at 13,000 rpm for 15 min at 4 °C, and the supernatants were collected. Protein concentration was determined using the Bradford assay [53]. Proteins (15–20 μg) were separated by sodium dodecyl sulfate-polyacrylamide gel electrophoresis (SDS-PAGE) and transferred to PVDF membranes. After blocking with 5% nonfat milk for 1 h in TBS-T, the membrane was incubated with primary antibodies overnight. The next day, the membrane was washed thrice for 10 min each with TBS-T buffer and incubated with secondary antibodies for 60 min. The membrane was then washed thrice for 10 min each with TBS-T buffer. The protein bands were detected using enhanced chemiluminescence (ECL). β-actin was used as an internal reference. The enhanced chemiluminescence detection system (LAS 500; GE Healthcare Bio-Sciences, Uppsala, Sweden) visualized the protein expression and quantified using Image J software (version 1.54).

#### 4.2.5. RT-PCR Analysis

Total RNA was extracted from the whole brain tissue of randomly selected C57BL/6J mice in each group using the easy-BLUE reagent (iNtRON Biotechnology, Republic of Korea). The purified RNA was quantified with a NanoDrop spectrophotometer (Molecular Devices, Sunnyvale, CA, USA), and 2.0 µg of RNA was then reverse-transcribed into cDNA using the High-Capacity cDNA Reverse Transcription Kit (Applied Biosystems, ThermoFisher scientific, Waltham, MA, USA). PCR was performed using this cDNA as a template with the following primers: iNOS (forward 5′-GAATTCACAGCTCATCCGGT-3′, reverse 5′-ACATTGATCTCCGTGACAGC-3′), COX-2 (forward 5′-AAAGCCCTCTACAGTGACAT-3′, reverse 5′-CATCTAGTCTGGAGTGGGAG-3′), and GAPDH (forward 5′-ACCACAGTCCATGCCATCAC-3′, reverse 5′-CCACCACCCTGTTGCTGTAG-3′). The PCR protocol included a pre-denaturation at 96 °C for 3 min, followed by cycles of 96 °C for 1 min (denaturation), 60 °C for 15 s (annealing), 72 °C for 30 s (extension), and a final extension at 72 °C for 5 min. GAPDH served as the internal control to determine relative iNOS and COX-2 expression levels, and the PCR products were analyzed on 1% agarose gels.

#### 4.2.6. Immunocytochemical Staining

To examine the anti-inflammatory effects of the CF, the translocation of NF-κB to the nucleus was detected by immunocytochemistry staining using a fluorescence microscope (Nikon ECLIPSE Ts2, Tokyo, Japan). BV-2 microglia cells were pretreated with the CF for 1 h, then LPS was added for 30 min. The cells were washed with PBS, fixed with acetone for 5 min, and then washed thrice with PBS. The BV-2 microglia cells were incubated with anti-NF-κB-p65 antibodies overnight at 4 °C. The secondary antibody labeled with Alexa Fluor 568 was treated at RT for 1 h. Nuclei were stained with DAPI (1 μg/mL) for 10 min. After washing, the stained cells were mounted and visualized using a fluorescence microscope and processed using NIS-Elements (BR-2.01.00, NY 11747–3064, NY, USA).

### 4.3. In Vivo Study

#### 4.3.1. Animals Handling and Treatment

Forty-eight male C57BL/6J mice (8 weeks old, 22 ± 1.5 g) were purchased from Daehan Bio-Link, Korea. Then, the mice were randomly divided into six groups of eight and housed in temperature-controlled conditions (12h light/dark cycle). All animals had free access to food and water. The animal experiments were performed according to the Institutional Animal Care and Use Committee (IACUC) guidelines of Konkuk University (IACUC approval number: KU25021). Study design and experimental groups LPS were freshly prepared in sterile saline before injection. Grouping was performed as follows: Group 1, Control: mice received only saline instead of either LPS or the extract; Group 2, LPS: mice received LPS (0.25 mg/kg/d; i.p.) during the 7 days of the second week. Groups 3, 4, and 5: the CF-treated mice received 25 mg/kg/d, 50 mg/kg/d, and 100 mg/kg/d of the CF by oral administration (p.o.) for 14 days, as well as LPS (0.25 mg/kg/d; i.p.) during the 7 days of the second week. Groups 6, donepezil treated: the mice received donepezil (DNZ) (5 mg/kg/d; p.o.) for the 14 days, as well as LPS (0.25 mg/kg/d; i.p.) during the 7 days of the second week.

Following behavioral assessment, the animals in each treatment group were anesthetized, and blood was taken from the heart and skull, which was rapidly removed, and the whole brain was collected. Serum was obtained from the blood by centrifuging (13,000 rpm, 30 s) the blood collection tubes (BD Microtainer, Lakewood, NJ, USA).

#### 4.3.2. Morris Water Maze (MWM) Test

The MWM test was carried out using the circle pool (60 cm depth and 150 cm diameter) half full of water (27 ± 1 °C). Briefly, the procedure included five consecutive training days and one test day. For 5 days of MWM behavioral training, if a mouse could not find the platform within 60 s, it was placed on the visible platform for 15 s. MWM behavioral tests were conducted using a hidden platform 1 h after the administration of CF (25, 50, and 100 mg/kg/d; p.o.) or DNZ (5 mg/kg/d; p.o.), as well as after the administration of LPS (0.25 mg/kg/d; i.p.) on the last day, Day 14. On the behavioral test day (Day 14), the distance traveled and the time spent finding the platform were assessed and compared between groups. For each mouse, the latency to find the platform and the distance traveled were recorded for up to 60 s using a video-tracking system. The data were analyzed using SMART 3.0 (Harvard Apparatus, Holliston, MA 01746, USA)

#### 4.3.3. Y-Maze Test

The Y-maze test was conducted to evaluate spontaneous alternation. Spontaneous alternation is based on the natural tendency of mice to avoid entering the same arm consecutively. Each arm of the maze was 38 cm long, 12 cm high, and 5 cm wide and converged to an equal angle. For 5 days of Y-maze behavioral training, each mouse was positioned at the center of the apparatus and allowed to explore the maze for a 5 min session. Y-maze behavioral tests were conducted after a specific rest period following the MWM test, which was performed 1 h after the administration of CF (25, 50, and 100 mg/kg/d; p.o.) or DNZ (5 mg/kg/d; p.o.), as well as after the administration of LPS (0.25 mg/kg/d; i.p.) on the last day, Day 14. Each mouse was positioned at the center of the apparatus and allowed to explore the maze for a 5 min session. The total number of entries and alternations was recorded during this time. The alternation percentage was determined by calculating the ratio of actual alternations to possible alternations, defined as (Total number of alternations/Total number of arm entries − 2) × 100%.

### 4.4. Statistical Analysis

All experiments were carried out in triplicate. In the case of animal experiments, behavioral tests were conducted on the final day, Day 14, and repeated three times within the same day. Other experiments were conducted over multiple days. Values are shown as means ± standard deviation (SD), and they were analyzed by analysis of variance (one-way ANOVA), followed by Dunnett’s test to determine the significance of differences using GraphPad Prism 5 (Graph Pad Software Inc., San Diego, CA, USA), *p* < 0.05 was considered statistically significant.

## 5. Conclusions

In this study, we demonstrated that CF derived from the ethanol extract of *V. vinifera* root exhibited neuroprotective and anti-neuroinflammatory effects against LPS-induced BV-2 microglia cells and the C57BL/6J amnesic mouse model. Our in vitro data indicates that CF concentration-dependently exerts anti-inflammatory effects by reducing NO production in BV-2 microglia cells induced by LPS. Further, CF suppresses inflammatory responses by decreasing the levels of iNOS and COX-2 protein expression and reducing the levels of proinflammatory cytokines. In addition, CF significantly suppresses the phosphorylation and degradation of IκB-α and inhibits NF-κB/IRF-3/MAPK signaling activation in LPS-activated BV-2 microglia cells. For in vivo study, CF administration improves spatial learning and cognitive function in mice with LPS-induced amnesia. Additionally, CF significantly reduced levels of proinflammatory cytokines (IL-6 and TNF-α) in the serum and the expression of inflammatory mediator proteins (iNOS and COX-2) in the whole brain of LPS-induced amnesic mice. Overall, our in vitro and in vivo data indicate that the CF derived from *V. vinifera* root ethanol extract may serve as a neuroprotectant during LPS-induced neurotoxicity.

## Figures and Tables

**Table 1:** Chemical composition of chloroform fraction (CF) from *V. vinifera* roots analyzed by LC-MS.

RT (min)	*m*/*z* ([M + H]^+^)	Formula ([M + H]^+^)	Compound
7.71	453.1317	C_28_H_21_O_6_	Viniferifuran
8.49	267.1558	C_11_H_13_O_4_	Ethyl caffeate
9.38	249.1113	C_14_H_17_O_4_	Prenyl caffeate
11.18	455.1469	C_28_H_23_O_6_	Maximol A or Pallidol
12.90	907.2713	C_56_H_43_O_12_	H-ampelopsin or viticanol B or viticanol C or Vitisin A or Vitisin C or Hopeaphenol

## Data Availability

The data associated with this research are available and can be obtained by contacting the corresponding author.
